# Enhancing adversarial resilience in semantic caching for secure retrieval augmented generation systems

**DOI:** 10.1038/s41598-026-36721-w

**Published:** 2026-02-11

**Authors:** Mohanad Afiffy, Mohamed Waleed Fakhr, Fahima A. Maghraby

**Affiliations:** 1 College of Engineering and Technology, Arab Academy for Science and Technology, Cairo, Egypt; 2 College of Computing and Information Technology, Arab Academy for Science and Technology, Cairo, Egypt

**Keywords:** Engineering, Mathematics and computing

## Abstract

Large Language Models (LLMs) combined with Retrieval-Augmented Generation (RAG) frameworks greatly improve natural language processing performance, but they incur substantial computational overhead because many similar queries are processed repeatedly. To mitigate this, semantic caching has been introduced to store past responses and reuse them for semantically similar inputs, thereby reducing computation costs. Yet, semantic caching mechanisms that depend only on semantic similarity are vulnerable to adversarial exploitation: carefully engineered malicious queries with minor lexical variations can trigger incorrect cache hits, undermining both the reliability and the security of the system. This paper examines security vulnerabilities in semantic proximity caching systems such as GPTCache, a widely used open-source semantic cache that exemplifies these issues, and introduces a new approach called SAFE-CACHE, which is built to withstand adversarial attacks. SAFE-CACHE adopts a cluster-centroid-based caching strategy that is fundamentally distinct from GPTCache’s single-query embedding method. It uses unsupervised clustering of historical query–answer pairs, statistical detection of noisy clusters, bi-encoder–based refinement, and conditional cluster enrichment driven by a fine-tuned lightweight LLM to infer the underlying intent of cached queries. During runtime, incoming queries are compared to cluster centroids instead of individual cached entries, enabling stronger semantic validation and improved resilience against adversarial behavior. Our experimental evaluations demonstrate that SAFE-CACHE dramatically reduces adversarial attack success rates from 52.77% to 14.27% compared to GPTCache, representing up to 72% improvement in adversarial resistance.

## Introduction

Retrieval-augmented generation (RAG) has emerged as a key technique for enhancing LLM outputs by incorporating relevant external knowledge directly into prompts. In a standard RAG workflow, user queries are supplemented with retrieved chunks from a knowledge base, allowing the model to generate responses that are more factual and contextually grounded. Modern RAG implementations also use semantic caching to further improve efficiency—caching the results of previous queries and reusing them when new semantically similar queries are issued. This strategy has seen widespread industry adoption, with major cloud providers such as Microsoft and Google integrating these capabilities into their platforms.in their Azure OpenAI services^1,2^ and Apigee API platform^3^, dramatically reducing latency and costs by avoiding repeated retrieval and LLM inference for redundant requests.

Yet these performance benefits introduce new security risks. In multi-user or untrusted settings, adversaries can misuse semantic cache sharing by designing queries that intentionally collide in the embedding space with legitimate queries. Such embedding collisions can cause the cache to return incorrect responses, which may expose sensitive information or allow malicious entries to be stored. Recent research has shown that LLM systems with shared caches are susceptible to information leakage and cache-poisoning attacks triggered by subtle changes to user inputs.

This work focuses on the fundamental issue that current semantic caching strategies for RAG systems are not robust to adversarial behavior. Although semantic caches greatly enhance efficiency, they provide insufficient protection against malicious queries or timing side-channel attacks that can undermine system integrity. This gap is critical: as organizations deploy LLMs in security-critical or privacy-sensitive contexts (such as enterprise chatbots or confidential digital assistants). In such scenarios, an insecure cache can become a primary vulnerability, allowing attackers to exfiltrate sensitive data or manipulate model outputs. Consequently, there is an immediate need for a caching approach that preserves the benefits of semantic reuse while also defending effectively against adversarial threats.

This research focuses on three main objectives. First, we develop a comprehensive adversarial attack methodology for stress-testing semantic caching systems, using MetMap metamorphic transformations and GPT-4.1 semantic attacks to produce hard adversarial samples designed to create embedding collisions. Second, we examine vulnerabilities in GPTCache and its industrial implementations (Microsoft Azure^1,2^ and Google Apigee^3^), which share the same single-query embedding matching architecture and serve as our baseline. Third, we introduce SAFE-CACHE, a cluster-centroid based semantic caching mechanism for intent classification that demonstrates enhanced security without significant performance degradation.

### Contributions

This work makes the following key contributions to secure semantic caching for RAG systems:

- Adversarial Attack Model: we formalize a comprehensive black-box adversarial attack model combining metamorphic query generation with realistic threat assumptions, including probing failure analysis and flooding strategies. This model functions as a benchmark for assessing the efficacy of defenses against cache poisoning attacks as well as a framework for evaluating security.
- SAFE-CACHE architecture: we propose SAFE-CACHE, a novel cluster-centroid based semantic caching mechanism that fundamentally differs from GPTCache’s single-query approach. The system employs Louvain community detection for Q&A pair clustering, statistical noisy cluster detection (purity and coherence criteria), bi-encoder refinement with adversarial triplet loss, and cluster-centroid matching at runtime to provide robust adversarial resistance while maintaining caching efficiency.
- Lightweight LLM for cluster enrichment: we develop and fine-tune a lightweight 1B-parameter Gemma-3 model using Low-Rank Adaptation (LoRA) for intent-preserving paraphrase generation. This model enables conditional cluster augmentation, generating high-fidelity training examples for sparse clusters while maintaining 98.3% JSON schema adherence and 94.8% intent preservation rate, ensuring robust cluster centroids and improved semantic matching without requiring extensive manual annotation.

### Related work

Efficiently handling repeated queries without repeatedly re-running the full Retrieval-Augmented Generation (RAG) pipeline particularly the LLM generation step—has become increasingly important. This need has driven growing interest in semantic caching, where systems reuse past computations instead of regenerating responses from scratch. In this section, we survey prior work across three central themes: approaches to semantic caching in RAG systems and modern LLMs, known vulnerabilities in vector-based semantic caches, and clustering-driven methods for semantic organization and data augmentation.

#### Semantic caching in RAG framework and LLM frontier models

Even before the advent of large language models (LLMs), semantic caching was explored as a means to accelerate question answering and query processing tasks. Luo et al.^4^ describe a semantic caching technique in the context of database query processing that stores the results of previous queries along with their semantic descriptions. When a new query arrives, the system can determine if its answer (or a portion of it) is already covered by the cached results, thus avoiding reprocessing the query from scratch. This early work, originating in pre-LLM QA systems, highlights how caching based on query semantics can significantly reduce redundant computations and improve response times.

Fu Bang (2023) developed GPTCache, employing embedding-based caching to reduce the computational cost associated with frequent LLM calls^5^. GPTCache utilized sentence-transformers for query embedding and vector databases (Milvus^6^, Weaviate, FAISS^7^) for similarity-based retrieval. This approach represents a significant advancement in framework-level caching, allowing systems to bypass LLM invocation entirely upon cache hits, thereby reducing both latency and computational overhead.

#### Relation to existing semantic cache implementations

Recent industrial implementations of semantic caching—including Microsoft’s Azure OpenAI semantic caching optimization^1^, Azure API Management semantic cache lookup policy^2^, and Google Cloud’s Apigee semantic caching policies^3^—follow the same underlying mechanism as GPTCache: *single-query embedding matching using vector similarity against previously cached requests*. Although the deployment contexts differ (framework-level cache vs. API gateway integration), these systems are architecturally equivalent to GPTCache in how they compute embeddings, store vectors, perform ANN lookup, and apply a static similarity threshold to decide cache hits. Consequently, all of these semantic caches share the same vulnerability class: adversaries can craft embedding-collision queries that satisfy the similarity threshold despite being semantically different. SAFE-CACHE does not target a specific implementation but instead addresses the common design assumption shared across these systems, namely that vector similarity alone is insufficient for semantic verification.

Ou et al. (2025) introduced Lookahead Cache Filtering (LCF), addressing efficiency in LLM inference by dynamically predicting and scheduling crucial key-value pairs^8^. LCF combines several mechanisms—important key-value lookahead prediction, Approximate Sorting, and Gather-based Matrix Multiplication—to reduce GPU memory use during inference without sacrificing output quality. It also incorporates Multi-Scale Pyramid Information Fusion, which strengthens the model’s ability to maintain coherence across long contexts. This design helps counter the “lost-in-the-middle” issue and preserves inference accuracy beyond what existing methods achieve. Rather than operating at the level of framework-wide caching, LCF focuses on optimizing the internal state management of the LLM itself during inference.

#### Vulnerability of using vector search in semantic caching

Recent studies have highlighted critical vulnerabilities in vector-based semantic caching systems. Wang et al.^9^ demonstrated that vector databases, which serve as the backbone of RAG caching mechanisms like GPT-Cache, can be exploited through adversarial queries—inputs that maintain structural similarity to benign queries but incorporate subtle lexical modifications that alter their semantics. These modifications can occur at the character, word, or multi-level stages^10^, leading to what they term “vector database poisoning.”

The MetMap paper^9^ specifically revealed that GPTCache and similar embedding-based caching systems remain vulnerable to adversarial manipulations where queries maintain structural similarity but differ semantically. Such adversarial attacks may lead to false cache hits, ultimately compromising the system’s reliability and potentially exposing sensitive information or providing incorrect responses to users.

These vulnerabilities stem from a core limitation of pure vector-similarity methods: they depend almost entirely on embedding distances while overlooking the actual semantic intent and contextual cues expressed in a query. This disconnect between surface-level syntactic similarity and true semantic equivalence leaves room for adversarial manipulation, underscoring the need for caching systems that incorporate deeper semantic validation beyond simple vector matching. Recent work has pushed these risks further. Qiu et al.^11^ introduced QEAttack, which uses genetic optimization combined with locality-sensitive hashing and dual-gradient fusion to sharply reduce query requirements while still preserving semantic similarity. Their results show that attackers can craft semantically coherent adversarial queries with very few optimization steps. Similarly, Yang et al.^12^ show that adversarial prompt and fine-tuning attacks can threaten even specialized domain models, emphasizing the broad applicability of adversarial techniques across different LLM applications.

#### Clustering and data augmentation for semantic understanding

The development of effective clustering and data augmentation techniques has been crucial for semantic understanding in natural language processing. Rebelo (2022) introduced an automatic update strategy for chatbot systems in utility companies, leveraging unsupervised clustering to uncover hidden customer intents^13^. This method utilized Azure-based NLP services, including stemming, vectorization, and dimensionality reduction via PCA and LSA, effectively clustering and retrieving missed customer queries. This study demonstrated how clustering can automatically discover semantic patterns without manual annotation.

Zhong et al. (2025) proposed a Clustering, Labeling, then Augmenting framework designed to enhance semi-supervised text classification tasks significantly^14^. Their method innovatively used clustering to identify representative “landmarks” for manual labeling, which subsequently served as high-quality intermediaries for generating synthetic labeled data via Retrieval-Augmented Generation (RAG), LLM-based rewriting, and synonym substitution. This approach reduced reliance on extensive human labeling and improved model accuracy by augmenting data quality and diversity, demonstrating the effectiveness of LLM-based augmentation for enriching sparse clusters.

#### Clustering and community detection

Blondel et al. (2008) introduced the Louvain algorithm for fast community detection in large networks^15^, which has since become a cornerstone method for modularity optimization in network clustering. The algorithm’s efficiency and effectiveness in identifying natural community structures make it particularly suitable for large-scale text clustering applications.

Hegde et al. (2025) explored the application of the Louvain algorithm for community detection in semi-supervised learning contexts^16,17^. Their approach utilized community detection algorithms to assign labels in peer-to-peer lending datasets, demonstrating how graph-based clustering methods can effectively group semantically related data points for improved predictive accuracy. This work validates the effectiveness of community detection approaches for organizing complex datasets into meaningful clusters.

Beniwal et al. (2019) demonstrated the effectiveness of community-based approaches for text clustering, showing how community detection algorithms can be successfully applied to document grouping tasks^18^. Their work provides empirical evidence that network-based clustering methods can effectively capture semantic relationships in textual data, supporting the application of such methods in semantic caching systems.

Table 1 summarizes their objectives, key techniques, and relevance to SAFE-CACHE to provide a structured comparison of the related approaches.

**Table 1:** Summary of related work in semantic caching, adversarial robustness, and intent understanding.

Authors/year	Method	Target problem	Key technique	Relevance to SAFE-CACHE
**Semantic caching approaches**				
Luo et al.^4^ (2003)	Semantic caching for DB queries	Avoid redundant computation in QA systems	Query semantic matching and result reuse	Early foundation demonstrating semantic caching benefits
Fu Bang^5^ (2023)	GPTCache	Reduce LLM computation cost in RAG	Embedding-based vector similarity with FAISS^7^/Milvus^6^	Primary baseline for vector-based caching vulnerabilities
Microsoft^1,2^ (2024–2025)	Azure Semantic Cache	Reduce cost and latency for OpenAI workloads	Single-query embedding lookup in API gateway or SDK	Architecturally identical to GPTCache; equally vulnerable to embedding-collision attacks
Google^3^ (2025)	Apigee Semantic Cache	API gateway caching for LLM workloads	Vector similarity-based cache lookup	Same single-query matching vulnerability as GPTCache and Azure
Ou et al.^8^ (2025)	Lookahead Cache Filtering (LCF)	Reduce GPU memory during LLM inference	KV prediction + approximate sorting + pyramid fusion	Internal LLM optimization; complementary to framework-level caching
**Adversarial vulnerabilities**				
Wang et al.^9^ (2024)	MetMap testing framework	Adversarial attacks on vector databases	Metamorphic transformations for vector DB poisoning	Core motivation for multi-layer semantic defenses
Qiu et al.^10^ (2022)	Adversarial NLP survey	Bypass similarity filters in caching systems	Multi-level query perturbations (char/word/sentence)	Demonstrates fundamental limits of pure vector similarity
**Clustering & data augmentation**				
Rebelo^13^ (2022)	Auto-update chatbot strategy	Hidden pattern discovery in customer queries	Unsupervised clustering + Azure NLP services	Supports Q&A pair clustering strategy and monitoring
Zhong et al.^14^ (2025)	Clustering-Labeling-Augmenting	Semi-supervised text classification	Cluster-based labeling + RAG-based augmentation	Validates clustering + LLM-based augmentation pipeline approach
Hegde et al.^16^ (2025)	Louvain-based label assignment	Community detection for semi-supervised learning	Community detection algorithms for grouping	Validates community detection for semantic clustering
Blondel et al.^15^ (2008)	Louvain algorithm	Fast community detection in large networks	Modularity optimization for network clustering	Core algorithm for Q&A pair community detection
Beniwal et al.^18^ (2019)	Community-based text clustering	Document clustering via network analysis	Community discovery for text grouping	Demonstrates community detection effectiveness for text

### Background

#### Large language models (LLMs)

Large language models have revolutionized Natural Language Processing (NLP) by enabling the generation of human-like text. However, their performance can be limited by the static nature of their training data, often lacking up-to-date information or specific details required for accurate user inquiries. RAG frameworks present a solution by combining the generative capabilities of LLMs with active information retrieval systems. The generation module then synthesizes the data retrieved to produce coherent contextual outputs^19^

#### Retrieval-augmented generation (RAG) architectures

**Fig. 1:**
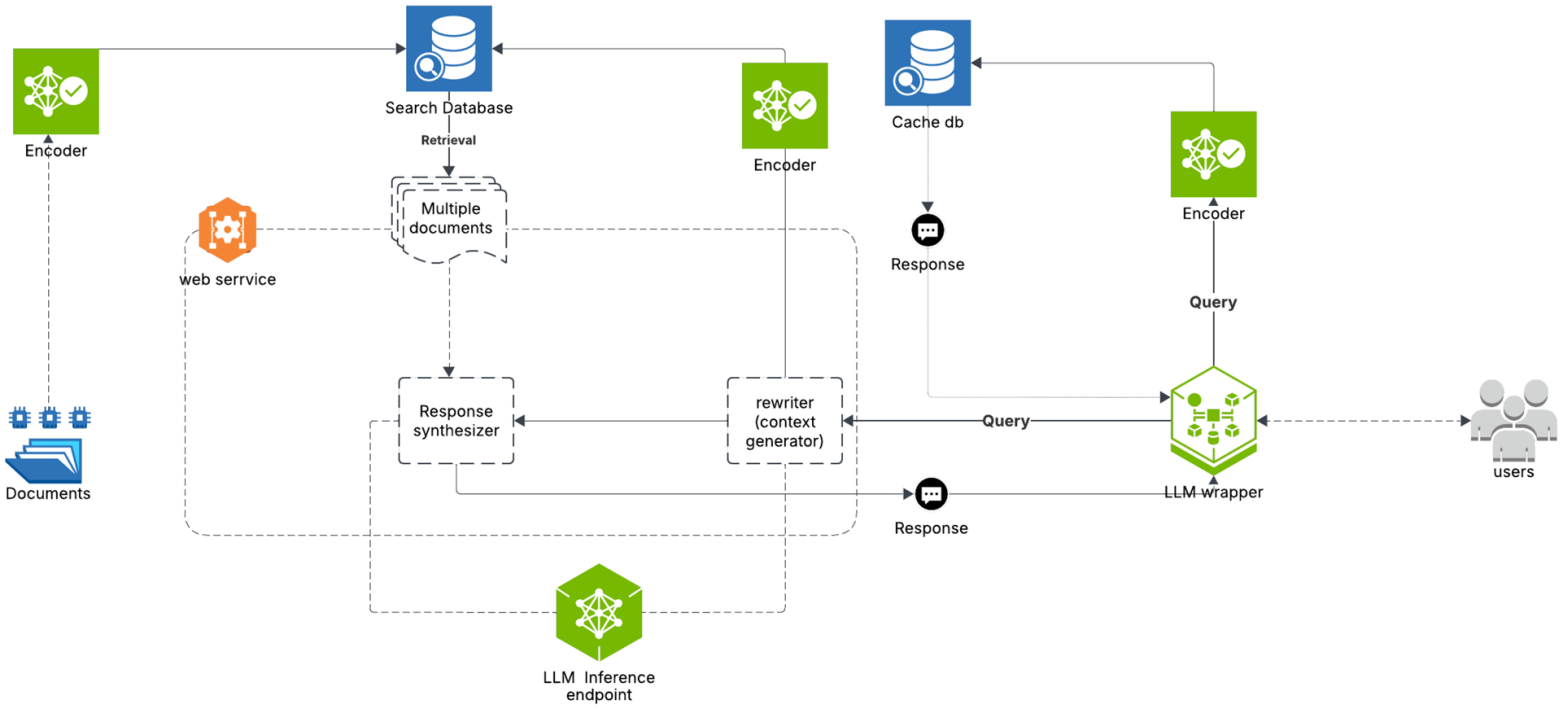
Overview of a typical retrieval-augmented generation (RAG) framework with integrated semantic caching. The system checks the semantic cache for similar queries before proceeding with document retrieval and LLM generation. Retrieved context from an external knowledge base is combined with the original query and passed into a generative model, while responses are cached for future semantic similarity matching.

The growing importance of RAG has driven the development of increasingly sophisticated strategies aimed at improving system performance and adapting models to specific application needs. For example, Jiang et al. introduced context-compression methods that make more efficient use of computational resources, allowing models to process larger and richer contextual information without compromising performance.^20^. In parallel, Quintela and Sapateiro emphasized a selective augmentation process that dynamically incorporates relevant information during inference, which can significantly enhance the relevance and accuracy of responses generated by RAG systems^21^. Domain-specific adaptations of RAG have also emerged, such as HybridRAG for aircraft fault diagnosis, which integrates knowledge graphs with multi-dimensional retrieval strategies combining graph-based reasoning with vector-based and BM25-based text retrieval to enhance diagnostic precision in specialized maintenance applications^22^. An illustration of a typical RAG framework with semantic caching architecture can be seen in Figure 1.

#### Semantic caching for RAG

One of the primary approaches to optimizing the cost and performance of RAG systems is through semantic caching, which addresses the computational overhead associated with repeated processing of similar queries within the RAG pipeline. Unlike traditional caching systems that rely on exact matches, semantic caching maintains a repository of previously processed questions and employs similarity measures to identify semantically equivalent queries, then return the cached response^1^.

The semantic caching process works by creating vector embeddings for incoming queries and comparing them to those that are already cached, using similarity metrics like cosine similarity. When a new query comes in, the system assesses how similar it is to previously cached queries. If the similarity score is higher than a set threshold, the system retrieves the cached response directly, avoiding the more resource-intensive processes of retrieval and LLM inference within the RAG framework. This method can greatly reduce response time and operational costs, especially in situations where many queries are similar.

Semantic caching is particularly useful in RAG-driven enterprise chatbots, customer support systems, and FAQ services, where users frequently ask different versions of the same basic question. For example, queries like “What is your return policy?” and “How can I return an item?” would be recognized as similar, allowing the system to provide the same cached answer for both without having to run through the entire RAG process again. This efficiency becomes even more critical as the number of user interactions increases, leading to considerable savings in retrieval and LLM API costs while still ensuring high-quality responses and a positive user experience.

#### Benchmarking RAG systems

With the rapid development of Retrieval-Augmented Generation (RAG) architectures, evaluating their effectiveness has become increasingly important. This has led to the creation of the *Retrieval-Augmented Generation Benchmark (RGB)* dataset, designed to assess retrieval-augmented generation models based on their ability to generate accurate responses from retrieved evidence. Each entry in RGB consists of a *query, an answer, and a set of supporting (positive) and opposing (negative) passages*. Positive passages provide factual evidence supporting the answer, whereas negative passages contain related but non-supportive or misleading information. By structuring the dataset in this way, RGB serves as a comprehensive benchmark for evaluating both *retrieval effectiveness and generation accuracy*, ultimately aiding in the development of more reliable fact-based RAG systems^23^.

The RGB dataset evaluates the RAG framework based on

- Noise robustness: evaluates the model’s ability to handle noisy, irrelevant documents.
- Negative rejection: tests the model’s capability to reject queries when no correct information is available.
- Information integration: assesses the model’s ability to synthesize answers from multiple documents.
- Counterfactual robustness: detects and corrects factual errors in the generated responses.

By addressing these aspects, the RGB dataset provides a robust benchmark for evaluating the performance and reliability of RAG systems in diverse scenarios.

## Methods

### Adversarial attack model

To rigorously evaluate the resilience of semantic caching systems, we develop a comprehensive adversarial attack model that blends metamorphic query generation with realistic black-box threat assumptions. Our goal is to assess cache-level defenses rather than to engineer the most query-efficient attack. Using two datasets of 300 intents each, we generate multiple benign and adversarial variants through both MetMap metamorphic transformations and GPT-4.1-based semantic attacks—including entity substitution, numeric drift, polarity shifts, and broader semantic divergence. This process yields challenging adversarial samples specifically crafted to induce embedding collisions, the core failure mode underlying cache poisoning.

#### Threat model

We assume a realistic black-box adversary with no access to system internals. Through reconnaissance, the attacker identifies approximately 300 recurring intents as poisoning targets. Table 2 summarizes the attacker’s capabilities and constraints.

**Table 2:** Threat model: attacker capabilities and constraints.

Aspect	Description
Access level	Black-box (no system internals)
Can do	Submit queries, observe answers
Cannot do	Compute/observe embeddings
	Know similarity thresholds or clustering
	View cache-update scheduling
Observation	Distinguish cached vs. LLM-generated answers
Reconnaissance	Identify $\sim$300 recurring intents
Attack goal	Inject adversarial Q&A pairs
Success criterion	Any adversarial sample enters benign cluster

#### Attack objective

The attacker begins by identifying which intents are most valuable to compromise. For each target intent, they assemble a set of positive examples—benign queries that a normal user might naturally submit—together with deliberately crafted adversarial variants. The objective is to introduce adversarial Q&A pairs into the system so that, when future benign queries resembling the positive examples are issued, the cache returns the attacker’s injected answer instead of the correct one. For stress-testing our system, we consider poisoning to be successful as soon as any adversarial sample merges into a benign cluster, since a single contaminated entry can influence subsequent cache refresh cycles.

#### Attack strategy

The attacker first attempts targeted probing to discover perturbation directions that might trigger cache collisions. This approach fails because the system does not perform synchronous caching: repeated queries receive identical LLM-generated responses, but no immediate cache hits. This behavior breaks the assumptions required for query-efficient attacks such as QEAttack^11^, which depend on rapid hard-label feedback to guide optimization.

With targeted probing rendered ineffective, the attacker shifts to a flooding strategy. They inject 900 adversarial Q–A pairs (three per intent), introducing diverse word-level and sentence-level perturbations across temperatures $t \in \{1,2,3\}$. Contamination appears only after scheduled cache updates, reflecting the asynchronous nature of the system. Under realistic black-box constraints, this flooding approach offers the most reliable means of evaluating cache resilience.

**Table 3:** Examples of adversarial query transformations.

Original query	Positive variant	Adversarial variant
What position did Jason Semore hold at Valdosta State before returning to Georgia Tech?	What was Jason Semore’s position at Valdosta State before returning to georgia tech?	What role did Jason Semore take on at Valdosta State after leaving Georgia Tech?
When is the premiere of ’Carole King & James Taylor: Just Call Out My Name’?	What is the premiere date for ’Carole King & James Taylor: Just Call Out My Name’?	When was the premiere of ’Carole King & James Taylor: Just Call Out My Name’ canceled?
When will American students start taking digital SAT exams?	What is the year that American students are scheduled to begin taking the digital SAT exams?	In which year will students in Europe begin taking the digital SAT exams?
Where is the Super Bowl held in 2022?	In which location is the 2022 Super Bowl taking place?	Where is the Super Bowl held in 2023?
Who won the 2022 Nobel Prize for chemistry?	Who was awarded the Nobel Prize in chemistry for the year 2022?	Who received the 2022 Nobel Prize for physics?
The genre of the drama *“Good Sam”* is what?	What genre does the drama ’Good Sam’ belong to?	The category of the comedy ’Good Sam’ is what?
How tall is the Statue of Liberty including its pedestal?	What is the full height of the Statue of Liberty including the base?	How tall is the Eiffel Tower including its base?
What is the population of New Zealand as of 2023?	How many people live in New Zealand in 2023?	What is the population of Australia as of 2023?
Who directed the film ’Dune: Part Two’?	Who is the director of ’Dune: Part Two’?	Who composed the soundtrack for ’Dune: Part Two’?

#### Dataset construction and augmentation

SAFE-CACHE development uses two dataset families: GPTCache-based data for model training and RGB-based data for adversarial evaluation. We augmented the GPTCache corpus ($\sim$600 entries) to create training data for the bi-encoder and Gemma-3 models. For evaluation, we built two adversarial test sets from RGB^23^, each covering 300 intents. The first uses MetMap^9^ metamorphic transformations to generate benign and adversarial variants at different perturbation levels. The second employs GPT-4.1 to create more sophisticated semantic attacks through entity substitution, numeric perturbation, polarity inversion, and category substitution. Table 3 shows representative query transformations, while Table 4 summarizes all datasets.

**Table 4:** Dataset summary for training and evaluation.

Dataset	Source	Size	Purpose
Bi-encoder training	GPTCache augmented	1800 triplets	Adversarial refinement
Gemma-3 fine-tuning	GPTCache augmented	600 examples	Paraphrase generation
Benign evaluation	RGB (300 intents)	1800 Q&A pairs	Cache precision
Adversarial evaluation	RGB augmented	900 Q&A pairs	Attack success rate
Total evaluation	RGB combined	2700 Q&A pairs	Robustness analysis

#### Empirical vulnerability analysis

**Fig. 2:**
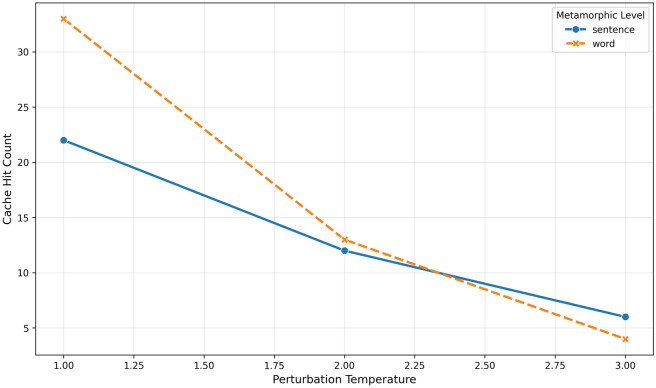
Cache behavior analysis on adversarial queries of different perturbation levels.

Experiments across perturbation temperatures identify the most vulnerable attack scenario. Figure 2 shows perturbation temperature 1 exhibits highest vulnerability, where subtle adversarial modifications achieve maximum cache hit rates. This reveals attackers can achieve optimal effectiveness with minimal query modifications, making detection extremely challenging.

### SAFE-CACHE architecture

#### Overview

**Fig. 3:**
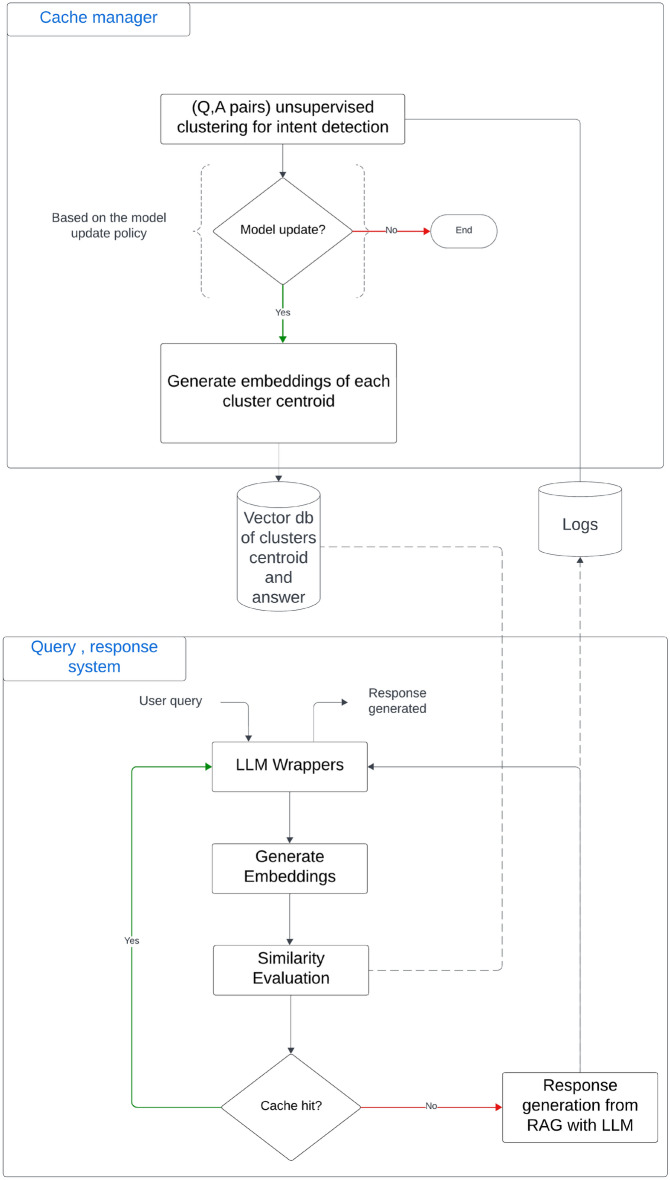
High-level overview of the SAFE-CACHE semantic caching system. Historical logs of queries and answers feed into unsupervised clustering, noisy cluster detection, bi-encoder refinement, and conditional cluster enrichment to build robust cluster representations. At query time, incoming queries are matched against cluster centroids; when similarity exceeds the threshold, cached responses are returned; otherwise, queries are forwarded to the LLM.

To address weaknesses in semantic proximity caching—particularly those stemming from vector‑database poisoning—we introduce SAFE‑CACHE, a semantic caching framework built around cluster centroids rather than single‑query embeddings. Unlike GPTCache’s direct embedding‑lookup design, SAFE‑CACHE organizes query–answer pairs into semantically coherent clusters and uses those clusters as the unit of caching. As illustrated in Fig. 3, the system operates in two phases: (1) *Offline Cache Manager*—processes historical query-answer pairs through clustering, noisy cluster detection, bi-encoder refinement, and conditional cluster enrichment to build robust cluster representations; and (2) *Runtime Query Response System*–incoming queries are compared against cluster centroids using similarity thresholds. If a centroid exceeds the threshold, the corresponding cached answer is returned; otherwise, the query is forwarded to the LLM. This architecture ensures that queries are assessed based on cluster-level semantic patterns rather than individual query matches, providing adversarial resilience while maintaining caching efficiency.

#### Cache manager

The offline Cache Manager processes historical query-answer pairs to build robust cluster representations through a multi-stage pipeline.

#### Q&A embedding

Our approach leverages combined question-answer embeddings rather than question-only embeddings. By incorporating response text alongside queries, we capture a richer semantic structure that helps distinguish between benign and adversarial interactions. For example, when examining two similar questions (*“Where is your shop safe?”* vs. *“Where is your shop?”*), considering the complete query-answer pairs reveals how the embeddings diverge significantly due to the distinct nature of their responses–one refusing to disclose sensitive information and the other providing helpful location details. This divergence naturally places such interactions in different clusters, providing inherent adversarial resistance. We use all-MiniLM-L6-v2^24^ as the base embedding model for the initial representation of the Q&A pair. Statistical validation of this design choice is presented in the Ablation Analysis section.

#### Initial clustering via Louvain community detection

Each question-answer pair is represented as a combined embedding using all-MiniLM-L6-v2^24^, forming a similarity graph where nodes represent embeddings and edges represent semantic closeness. We apply the Louvain method^15^, proven effective for identifying natural groupings of semantically related text documents^18^. Algorithm 1 (Phase 1) details the process: we build the similarity graph using a FAISS approximate nearest neighbor (ANN) index^25^ for efficient retrieval, then apply community detection to produce discrete clusters. The similarity threshold for edge construction was determined through bootstrap experiments (see Ablation Analysis). These clusters ideally correspond to distinct user intents^26^, each encompassing paraphrastic query variants mapping to the same response. However, initial clustering may produce noisy clusters containing adversarial contamination or mixed intents, necessitating refinement.

#### Noisy cluster detection

Following initial community detection, SAFE-CACHE identifies noisy clusters using two stability criteria (Algorithm 1, Phase 2):

(1) Purity score ($< 0.85$): the cluster’s purity is measured by comparing the dominant class proportion. A purity score below 0.85 indicates mixed or adversarial contamination:

$$Purity(C) = \frac{\max _k |C_k|}{|C|} < 0.85,$$

where bootstrap analysis shows clean clusters maintain 95% CI of [0.86, 0.92].

(2) Minimum intra-similarity ($< 0.85$): the minimum pairwise cosine similarity within the cluster must exceed 0.85 for semantic consistency. Clusters failing this criterion are semantically inconsistent:

$$\mu _{\text {sim}}(C) = \frac{1}{|C|(|C|-1)}\sum _{i\ne j}\cos (x_i,x_j) < 0.85.$$

**Fig. 4:**
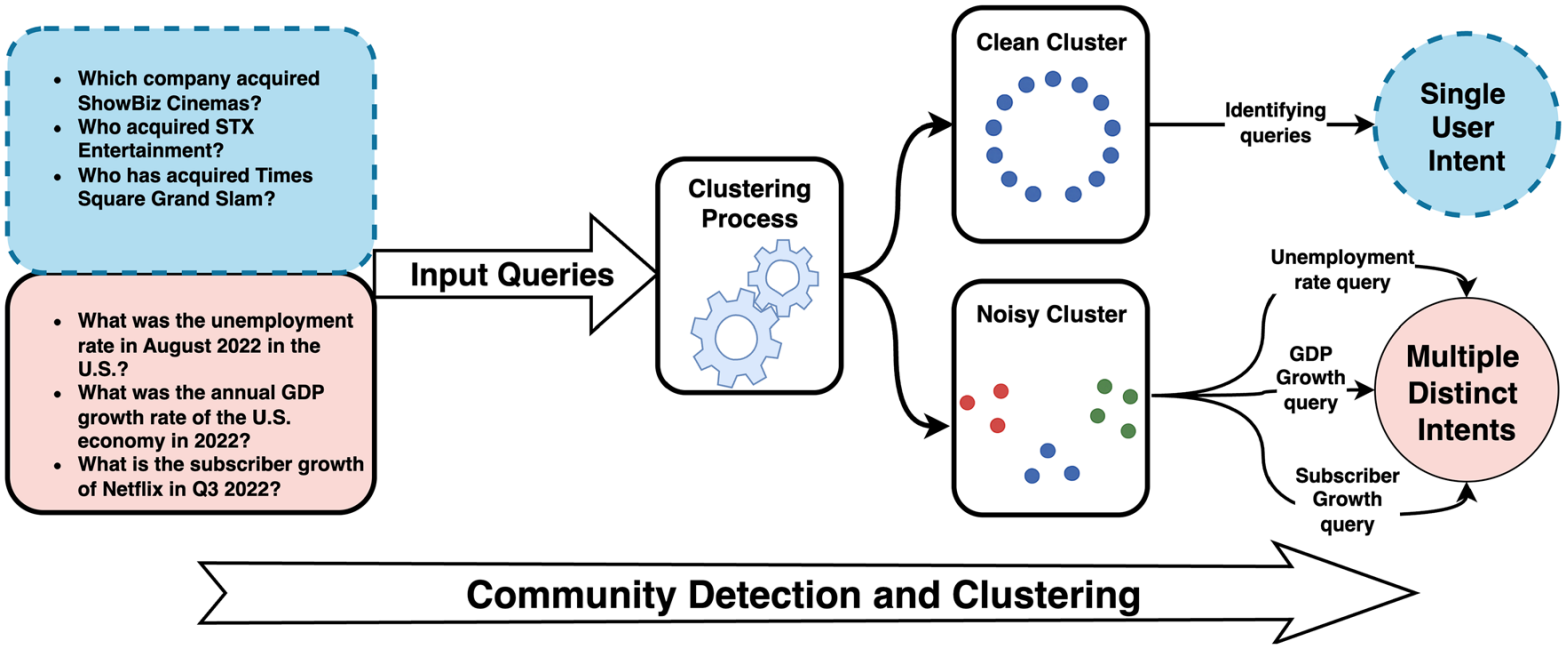
Community detection clustering results showing clean clusters (single coherent intent) versus mixed clusters (multiple distinct intents). The clustering process generates both types, with clean clusters facilitating straightforward intent extraction. In contrast, mixed clusters benefit from LLM’s ability to identify and extract multiple valid intents from the same cluster.

These two metrics—purity and intra-similarity—form the standard criterion for identifying noisy clusters requiring refinement. Figure 4 illustrates how community detection produces both clean clusters (single coherent intent) and mixed clusters (multiple distinct intents).

#### Bi-encoder refinement

Detected noisy clusters are refined using a fine-tuned bi-encoder (BAAI/bge-small-en-v1.5^27^) trained on approximately 1800 adversarial Q&A triplets derived from the GPTCache base dataset (600 entries augmented with LLM-generated positive and adversarial variants). The model uses combined loss:

$$L = L_{\text {triplet}} + \lambda _1 L_{\text {cosine}} + \lambda _2 L_{\text {direction}},$$

where

$$\begin{aligned} L_{\text {triplet}}= & \max (0, m + d(a,p) - d(a,n)),\\ L_{\text {cosine}}= & 1 - \cos (a,p),\\ L_{\text {direction}}= & -\log (1-\cos (a,n)+\epsilon ). \end{aligned}$$

This loss increases similarity among benign Q&A items while decreasing similarity with adversarial items. The fine-tuned bi-encoder re-embeds noisy cluster members, producing improved embeddings trained specifically on metamorphic transformations. We then rebuild a local similarity graph using the same threshold determined by bootstrap experiments and reapply Louvain community detection. The number of resulting sub-clusters is not predetermined–it emerges naturally from the semantic structure and similarity threshold, independent of query counts. This data-driven splitting ensures that cluster refinement is fully automatic and based solely on improved semantic representations.

Table 5 illustrates representative examples from selected clusters, demonstrating how the final cleaned clustering process naturally groups queries with shared semantic patterns to make every cluster as an intent.

**Table 5:** Examples of cluster-based semantic grouping with query–answer pairs.

Cluster ID	Example queries	Answer
cluster_585	Which company won Yahoo Finance’s 2022 “Company of the Year” Award?	Costco
	Who was named ’Company of the Year’ for 2022 by Yahoo Finance?	
	Which company was named Yahoo Finance’s 2022 “Company of the Year”?	
	Who was awarded the ’Company of the Year’ by Yahoo Finance in 2022?	
cluster_586	Who won the Arizona Senatorial Election 2022?	Mark Kelly
	Who was the victor in the 2022 Senate race for Arizona?	
	Who won the 2022 U.S. Senate race in Arizona?	
	Who was the victor in the 2022 Arizona Senatorial Election?	
cluster_587	How much does Tesla now charge for its Full-Self Driving (FSD) software?	$15,000
	How much does Tesla currently charge for its Full-Self Driving (FSD) software?	
	What is the new price of Tesla’s Full-Self Driving (FSD) software?	
	How much does Tesla’s Full-Self Driving (FSD) software cost now?	
cluster_588	Who is the founder of Midjourney?	David Holz
	Who founded Midjourney?	

#### Cluster monitoring

The monitoring system tracks variation in relative cluster sizes over time. We use relative frequency rather than absolute frequency because message counts vary considerably between clusters within a given period. We define the monitoring metric as:

1 $$\begin{aligned} d_{i(t)} = P_{i(t)} - P_{i(t_0)}, \end{aligned}$$

where $P_{i(t)}$ is the relative size of cluster *i* at time *t*, computed as the ratio of missed messages in cluster *i* to total missed messages at time *t*. The reference time $t_0$ corresponds to the last model update. The metric $d_{i(t)}$ is computed daily for all clusters; if the change exceeds a threshold, a model update is triggered^13^.

#### Conditional cluster enrichment

Following clustering and refinement, sparse clusters with fewer than 5 examples may produce unstable centroids as our goal that each cluster represents an intent. To address this, we employ conditional augmentation using a fine-tuned lightweight Gemma-3 1B model^28,29^ trained on approximately 600 paraphrase examples from the GPTCache base dataset. The model uses Low-Rank Adaptation (LoRA) with frozen backbone weights to enforce semantic conservativeness, achieving 98.3% JSON schema adherence and 94.8% intent preservation rate. Table 6 summarizes the model configuration and performance.

**Table 6:** Paraphrase generation model configuration and performance.

Parameter	Value
Base model	Gemma-3 1B (Google)
Adaptation method	LoRA (frozen backbone)
LoRA rank (*r*)	48
LoRA scaling ($\alpha$)	192
Training dataset size	$\sim$600 entries (GPTCache base)
Training epochs	2
Learning rate	$5 \times 10^{-5}$
JSON schema adherence	98.3%
Intent preservation rate	94.8%

#### Cluster centroid computation

After clustering, refinement, and enrichment, each cluster is represented by its centroid—the mean embedding of all Q&A pairs within that cluster. For cluster $C_i$ containing $n_i$ Q&A pairs with embeddings $\{{\bf u}_1, {\bf u}_2, \ldots , {\bf u}_{n_i}\}$, the cluster centroid is computed as:

$${\bf c}_i = \frac{1}{n_i}\sum _{j=1}^{n_i} {\bf u}_j$$

This centroid represents the semantic center of the cluster and serves as the reference point for runtime query matching. The enrichment process using the fine-tuned Gemma-3 model ensures that sparse clusters have sufficient examples to compute stable, representative centroids that accurately capture the cluster’s semantic intent.

Algorithm 1 provides the complete specification of the clustering and refinement pipeline.

#### Query response system

At query time, the system follows a cluster-centroid matching pipeline:

1. Query embedding: a new user query *q* is embedded using the same encoder to produce query embedding ${\bf v}_q$.
2. Centroid similarity matching: the system computes cosine similarity between ${\bf v}_q$ and all clean cluster centroids $\{{\bf c}_1, {\bf c}_2, \ldots , {\bf c}_k\}$:
  $$\text {sim}_i = \cos ({\bf v}_q, {\bf c}_i) = \frac{{\bf v}_q \cdot {\bf c}_i}{\Vert {\bf v}_q\Vert \Vert {\bf c}_i\Vert }$$
3. Cache lookup: if $\max _i(\text {sim}_i) \ge \tau _{\text {cluster}}$ (where $\tau _{\text {cluster}}$ is the similarity threshold), the system retrieves the cached response associated with the best-matching cluster, circumventing LLM invocation and significantly reducing latency and computational cost.
4. LLM fallback: if all similarities fall below the threshold, the query is deemed novel and proceeds to the retrieval-augmented generation process, where external knowledge sources are queried and the LLM generates the final answer.

**Algorithm 1:**
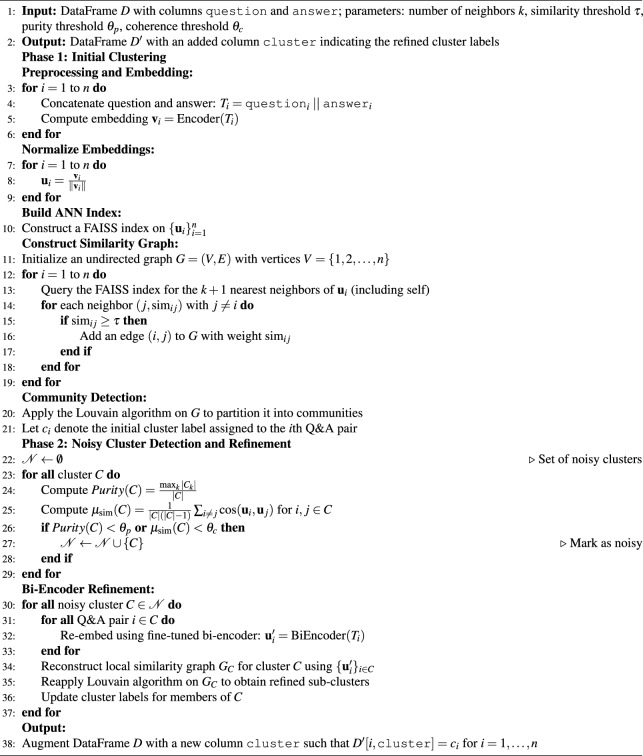
ANN-based graph clustering with noisy cluster refinement for Q&A pair data.

### Experimental setup

## Adversarial attack success rate (ASR) experiment

This section presents the experimental evaluation of adversarial success rates (ASR) for both GPTCache-equivalent behavior and SAFE-CACHE under the threat model formalized in section “Adversarial attack model”.

### Experiment design

#### Benign prefill

The semantic cache is prefilled with:

$$1800 \text { benign Q--A pairs representing } 300 \text { intents}.$$

#### Adversarial set

The attacker injects 900 adversarial Q–A pairs ( 3 per intent).

#### Evaluation metric: cluster-level ASR

Adversarial success is defined as:

$$\text {ASR} = \frac{\text {Number of adversarial Q--A entries assigned to benign clusters}}{\text {Total adversarial queries}}.$$

A single adversarial infiltration counts as a success because any contamination eventually compromises cluster purity and centroid representativeness during future refresh cycles.

We evaluate two systems:

1. GPTCache-equivalent: MiniLM embeddings.
2. SAFE-CACHE: Louvain clustering + BGE bi-encoder cleaning refinement.

### Results

#### Comparative security analysis

We evaluate adversarial resilience under two attack scenarios: (1) MetMap-based metamorphic transformations (word-level and sentence-level perturbations), and (2) GPT-4.1-generated semantic attacks (entity substitution, numeric perturbation, polarity/logical direction inversion, and category substitution). Table 7 presents the adversarial success rate (ASR) comparison between GPTCache (single-query embedding matching) and SAFE-CACHE (cluster-centroid based matching). GPTCache relies solely on vector similarity thresholds against individual cached queries without clustering or centroid-based verification, making it vulnerable to adversarial queries that achieve high embedding similarity despite semantic differences.

**Table 7:** Adversarial success rate (ASR) comparison: GPTCache vs. SAFE-CACHE.

System	MetMap ASR (%)	GPT-4.1 ASR (%)	Relative reduction
GPTCache (embedding-only)	52.77	59.2	–
SAFE-CACHE (cluster-centroid)	14.27	20.3	72.7% / 65.7%

SAFE-CACHE achieves a 72.7% relative reduction in adversarial success rate for MetMap attacks and 65.7% reduction for GPT-4.1 semantic attacks compared to GPTCache’s single-query matching approach. The GPT-4.1 attacks show approximately 6% higher ASR for both systems due to more sophisticated semantic manipulations (entity substitution, polarity inversion, category substitution) that better preserve embedding similarity while altering meaning. While GPT-4.1-based attacks incur significantly higher query costs in production environments, our evaluation focuses on adversarial resilience testing rather than query-efficient attack scenarios, which remain an important direction for future work. The substantial improvements across both attack types demonstrate that the multi-stage clustering, refinement, and cluster-centroid matching pipeline significantly enhances resilience against diverse black-box adversarial poisoning attempts.

### Computational overhead analysis

SAFE-CACHE’s computational overhead happens in the offline Cache Manager phase, with minimal impact on runtime query response latency. Table 8 shows the processing time for Cache Manager components, while Table 9 compares runtime query response latency.

**Table 8:** Cache manager offline processing time (1800 Q&A pairs).

Component	Operation	Processing time
Clustering	Louvain community detection	$\sim$24 s (total)
Noisy cluster detection	Purity + coherence analysis	Included in 24 s
Bi-encoder refinement	Fine-tuned BGE re-embedding	4s per cluster
Cluster enrichment	Gemma-3 1B paraphrase generation	$\sim$10 s per sparse cluster

**Table 9:** Runtime query response latency comparison.

Component	GPTCache	SAFE-CACHE
Query embedding	MiniLM-L6-v2	MiniLM-L6-v2
Similarity matching	Single-query cosine	Cluster-centroid cosine
Total latency impact	Baseline	No measurable difference

The Cache Manager operations run offline during scheduled cache updates and don’t affect user-facing query latency. Clustering and cleaning 1800 Q&A pairs takes about 24 s, showing minimal overhead for the core pipeline. The main computational cost is cluster enrichment using the fine-tuned Gemma-3 1B model, averaging 10 s per sparse cluster. Notably, this enrichment overhead can be eliminated entirely in production by configuring the system to only consider clusters that naturally accumulate $\ge 5$ examples through organic query traffic. In heavily-used RAG systems where repetitive queries are common, most clusters naturally reach this threshold without requiring synthetic augmentation, making enrichment optional. At runtime, SAFE-CACHE performs cluster-centroid matching using the same embedding model as GPTCache, resulting in comparable inference times. All experiments were conducted on Nvidia T4 GPU (15 GB VRAM).

### Ablation analysis

To validate our design choices, we tested how different components affect adversarial robustness.

#### Q&A vs. questions-only embedding comparison

We compared clustering using combined question-answer embeddings versus question-only embeddings on 2700 entries (1800 benign, 900 adversarial).

#### Pairwise separation analysis

**Fig. 5:**
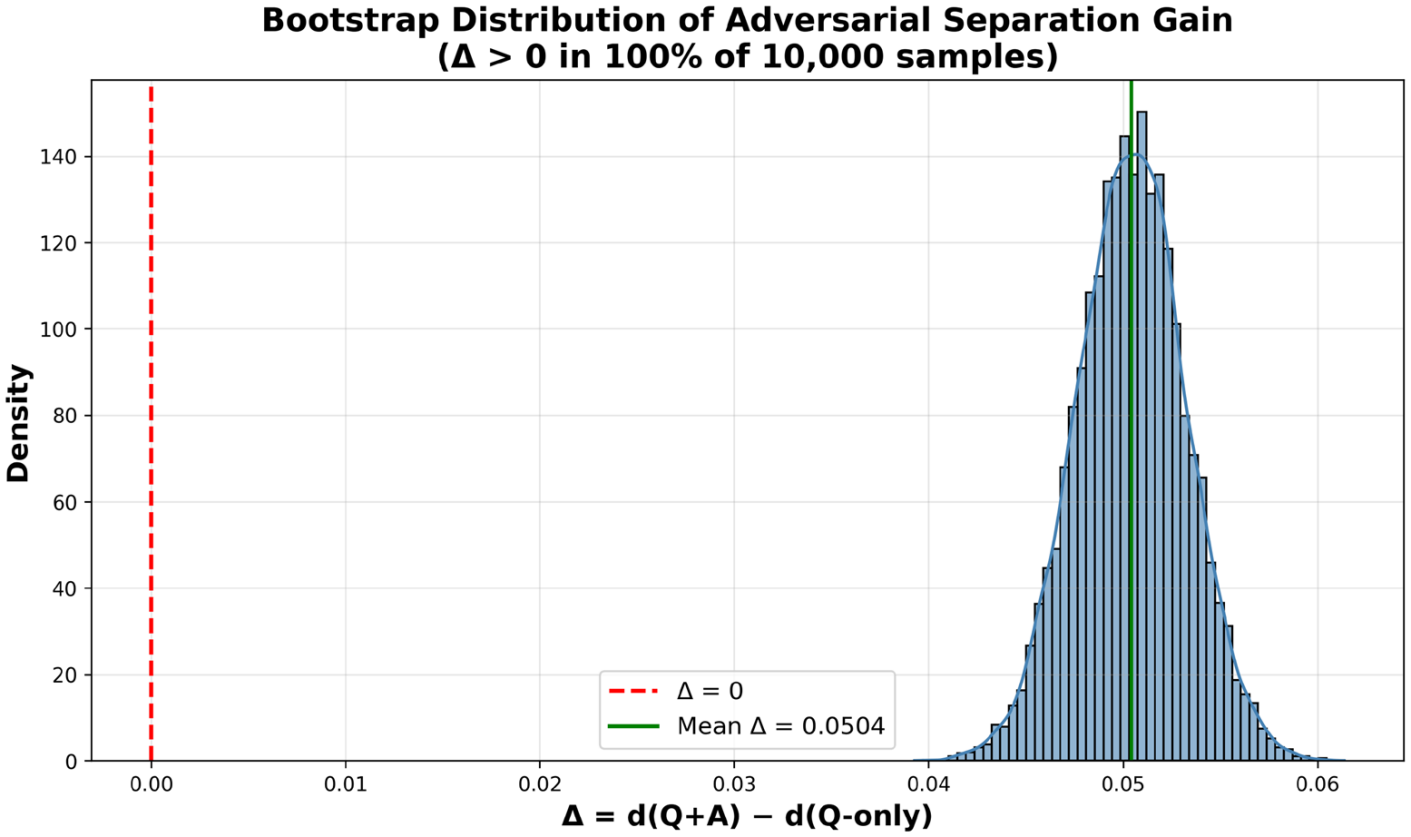
Bootstrap distribution of adversarial separation gain over 10,000 samples. All samples show positive separation (mean = 0.0504, Cohen’s $d = 0.57$), showing that Q&A embeddings improve adversarial separation compared to question-only embeddings ($p < 10^{-4}$).

Testing 1000 benign-adversarial pairs shows Q&A embeddings create better separation ($\Delta = d(Q+A) - d(Q\text {-only})$). All samples show positive separation (mean = 0.0504, Cohen’s $d = 0.57$, $p < 10^{-4}$), as shown in Fig. 5.

#### Clustering-level validation

**Fig. 6:**
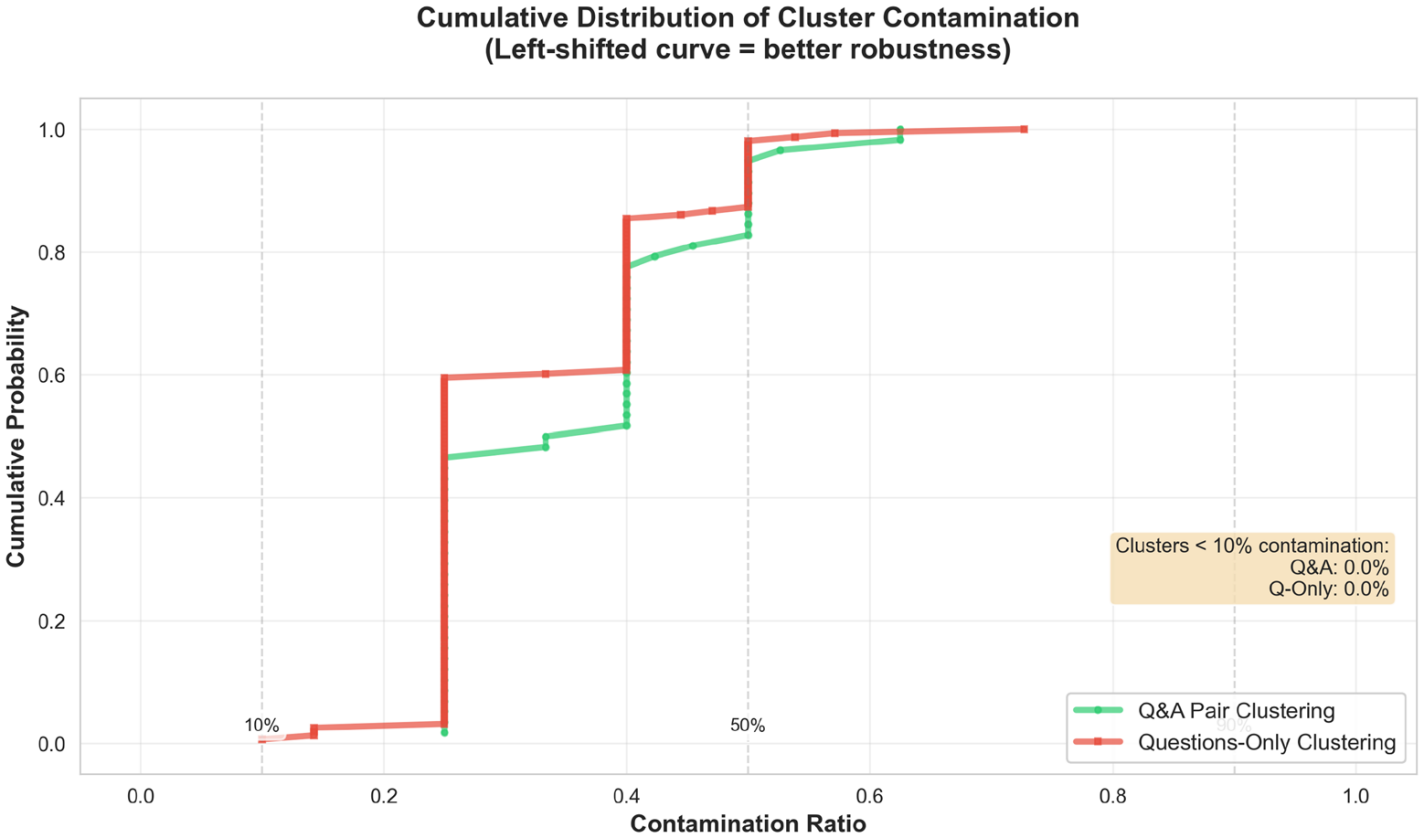
CDF of contamination ratios across contaminated clusters. Q&A clustering (solid) exhibits lower contamination severity versus questions-only (dashed), with median ratios of 0.33 vs. 0.41, limiting semantic poisoning extent.

Q&A clustering achieves 14.27% adversarial leakage versus 32.66% for questions-only—a 56.3% reduction (Table 10). The confidence intervals do not overlap ([12.1, 16.7] vs. [29.7, 35.8]), confirming stronger adversarial separation. Q&A clustering also reduces contamination severity (median 0.33 vs. 0.41), with 60% of contaminated clusters below 0.35 contamination ratio compared to 40% for questions-only (Fig. 6).

**Table 10:** Adversarial leakage comparison: Q&A vs. questions-only clustering.

Clustering method	ASR (%)	Leaked queries	95% CI	Contamination ratio
Q&A clustering	14.27	128/900	[12.1, 16.7]	0.33
Questions-only	32.66	293/900	[29.7, 35.8]	0.41

#### Paraphraser quality comparison: GPT-4.1 vs. fine-tuned Gemma-3 1B

We compared paraphrase quality between GPT-4.1^30^ (our baseline) and our fine-tuned Gemma-3 1B LoRA model, since paraphrase quality directly affects how well clusters work for semantic caching.

**Table:** 

**Evaluation Protocol:** 100 drift-sensitive queries across 7 domains. Each system produced 5–6 paraphrases, manually annotated for: intent fidelity, entity preservation, temporal precision, specificity preservation, repetition rate, diversity, and schema adherence.	

Quantitative results: Table 11 summarizes the performance comparison across seven evaluation metrics.

**Table 11:** Paraphrasing performance comparison

Metric	GPT-4.1	Gemma-3 1B
Intent Preservation	96.5	94.8
Entity Preservation	97.5	92.1
Temporal Precision	98.0	91.7
Specificity Preservation	93.0	**96.1**
Diversity (Distinct-2)	0.78	**0.83**
Repetition Rate	12.0	**8.5**
Schema Adherence	**100**	98.3

**Table:** 

**Key Finding:** The fine-tuned 1B model achieves better specificity preservation (+3.1pp: 96.1% vs. 93.0%) and diversity (+6%: 0.83 vs. 0.78) compared to GPT-4.1, while reducing repetition by 29%. Although GPT-4.1 shows stronger entity preservation (97.5% vs. 92.1%) and temporal precision (98.0% vs. 91.7%), the lightweight model’s superior specificity preservation and diversity make it well-suited for semantic caching where precise query semantics and varied paraphrases matter most, with significant cost savings.	

Qualitative analysis:

Representative correct behavior:

*Query:* “When was the Samsung Galaxy S23 officially announced?”

Paraphrases:

- “When did Samsung officially announce the Galaxy S23?”
- “What is the announcement date for the Samsung Galaxy S23?”

$\checkmark$ Preserves entities and temporal markers

*Query:* “What was the population of Tokyo in the 2020 census?”

Paraphrases:

- “How many people lived in Tokyo during the 2020 census?”
- “Tokyo’s population count in the 2020 census?”

$\checkmark$ Maintains “2020 census” temporal anchor

Failure mode analysis:

**Table:** 

**Gemma-3 1B: Specificity Loss**	
*Input:* “Who scored the winning goal in the 2014 FIFA World Cup Final?”	
$\times$ “Who was the goalscorer in the 2014 FIFA World Cup Final?”	
Drops “winning” $\rightarrow$ changes answer scope

**Table:** 

**GPT-4.1: Scope Shift**	
*Input:* “Who was the winner of the 2022 Citrus Bowl?”	
$\times$ “Who defeated their opponent in the 2022 Citrus Bowl?”	
Generalizes from winner to any decisive play	

The models exhibit complementary strengths: GPT-4.1 excels at preserving entities and temporal markers, while Gemma-3 1B provides better specificity preservation and diversity. For semantic caching, where the goal is generating varied paraphrases that maintain precise query semantics for cluster enrichment, the lightweight model’s strengths align well with system requirements while offering substantial computational and cost advantages.

#### Cluster centroid threshold optimization

We tested thresholds from $\tau = 0.50$ to 0.90 using five independent resamplings to find the best centroid similarity cutoff. For each threshold, we measured cache precision, attack success rate (ASR), and F$_1$ score (harmonic mean of precision and $1 - \textrm{ASR}$) with $95\%$ confidence intervals.

**Fig. 7:**
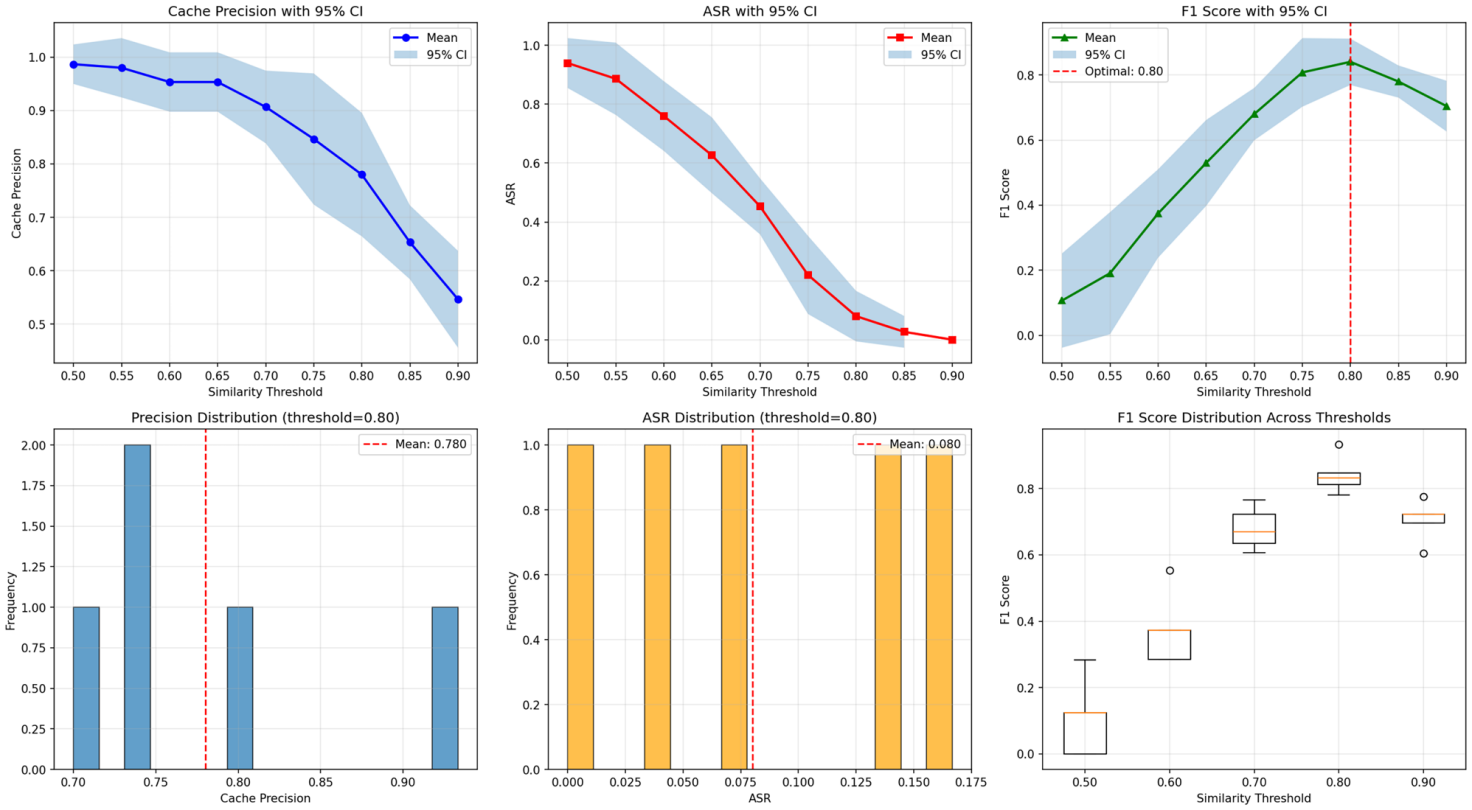
Threshold sweep analysis for cluster centroid similarity threshold $\tau \in [0.50, 0.90]$. The plot shows cache precision (blue), attack success rate (ASR, orange), and F$_1$ score (green) with $95\%$ confidence intervals across five resamplings. The F$_1$ score peaks at $\tau {=}0.80$, providing the best balance between cache utility and adversarial resistance. Boxplots (right panel) confirm low variance at this threshold, validating its robustness for production deployment.

As shown in Figure 7, cache precision drops as $\tau$ increases, while ASR shows the opposite pattern. The F$_1$ curve peaks at $\tau {=}0.80$, where cache precision reaches $78.0\%\ (\pm 9.3\%)$ and ASR stays low at $8.0\%\ (\pm 6.9\%)$. Both metrics differ significantly from random-matching baselines ($p{<}0.01$). Nearby thresholds ($\tau {=}0.75$ and $\tau {=}0.85$) show no significant drop ($p{>}0.05$), indicating a stable range. Note that this 8.0% ASR measures runtime centroid-matching success–how many adversarial queries get similar enough to cluster centroids to trigger cache hits. This differs from the 14.27% adversarial leakage rate reported earlier, which measures cluster-level contamination (any adversarial entry within benign clusters). Even when adversarial samples get into a cluster, they may not shift the centroid much if benign samples outnumber them, preventing adversarial queries from matching the centroid at query time.

We use $\tau {=}0.80$ as the default SAFE-CACHE threshold, providing the best balance between cache utility and adversarial resistance. All experiments used all-MiniLM-L6-v2 embeddings for consistency with GPTCache baseline. Note that this optimal threshold is specific to all-MiniLM-L6-v2; practitioners using SAFE-CACHE with different embedding models should run similar tests to find the optimal threshold for their chosen encoder.

#### Ablation summary

Our ablation studies validate three key design choices (Table 12): (1) fine-tuned Gemma-3 1B provides good paraphrase generation accuracy (96.1% specificity preservation, 0.83 diversity) with significant computational savings compared to GPT-4.1, running efficiently on T4 GPU (15 GB VRAM) without expensive API calls; (2) threshold optimization identifies $\tau = 0.80$ as optimal, achieving 78.0% precision with only 8.0% ASR; (3) Q&A embeddings reduce adversarial leakage by 56.3% compared to questions-only clustering. Together, these findings confirm that SAFE-CACHE’s multi-stage pipeline with lightweight models provides strong security without excessive computational complexity.

**Table 12:** Ablation studies summary.

Ablation focus	Variants compared	Key insight
Q&A vs. Q-only Embeddings	Combined vs. question-only	Q&A clustering reduces adversarial leakage by 56.3% (14.27% vs. 32.66% ASR) with lower contamination severity (0.33 vs. 0.41 median ratio)
Paraphraser Quality	GPT-4.1 vs. Gemma-3 1B LoRA	Gemma-3 achieves +3.1pp specificity preservation and +6% diversity with 29% lower repetition, validating lightweight model deployment
Centroid Threshold ($\tau$)	$\tau \in [0.50, 0.90]$ via bootstrap	Optimal $\tau = 0.80$ achieves 78.0% precision and 8.0% ASR with stable 0.75–0.85 plateau

## Discussion

This work examines security limitations in embedding-based semantic caching for Retrieval-Augmented Generation (RAG) systems and presents a multi-stage defense framework that improves adversarial resilience. Our findings show that caches using simple semantic proximity measures–such as cosine similarity between individual query embeddings–are vulnerable to metamorphic query perturbations. Even small lexical or structural changes can trigger false cache hits, making baseline semantic caches susceptible to leakage and poisoning.

SAFE-CACHE improves robustness by introducing a layered semantic validation pipeline that shifts caching from instance-level similarity to intent-level representation. The combination of community detection, noisy-cluster cleaning, bi-encoder refinement, and lightweight LLM-driven enrichment creates clusters that are semantically richer and more resistant to adversarial infiltration than single-vector baselines. A key finding is that using combined *question–answer* embeddings—followed by refinement and enrichment—provides much better separation between benign and adversarial samples. This multi-stage process not only increases cluster purity but also stabilizes centroid representations, making it much harder for adversarial queries to match with established intents.

Another important observation is that cluster enrichment using a fine-tuned lightweight LLM significantly increases semantic density within each cluster. By generating consistent, intent-preserving paraphrases, the enriched clusters produce centroids that are more robust against perturbation-driven misalignment. This shows that adversarial robustness is not just about embedding models, but can be systematically improved through curated cluster expansion and representation hardening.

At the same time, the study highlights opportunities for future improvements. Additional classification layers—such as Hybrid-DIET or other NLU-style intent models—may further strengthen robustness by introducing orthogonal decision boundaries that are harder for adversarial queries to bypass. However, integrating such models adds computational and latency overhead. A systematic comparison between the security gains and operational cost of these classifiers remains an important direction for future work, particularly in high-throughput or multi-tenant RAG deployments. In the current SAFE-CACHE design, we rely on cluster-centroid matching, which naturally handles ambiguous or multi-intent queries without requiring explicit multi-label classification. When a query expresses multiple intents or overlaps with several clusters, its embedding typically falls below the centroid similarity threshold for all existing clusters, routing it to the RAG pipeline for LLM processing. The resulting Q&A pair is stored in history and, during the next Cache Manager refresh, may form a new cluster representing the intent combination or attach to an existing cluster if similar mixed-intent queries accumulate. This makes SAFE-CACHE inherently robust to multi-intent queries: ambiguous inputs don’t force incorrect cache hits, and the clustering process automatically adapts as new query patterns emerge.

The security improvements shown here should not be interpreted as formal guarantees, but rather as strong empirical evidence that multi-layered semantic validation offers meaningful protection against black-box metamorphic attacks. SAFE-CACHE’s design follows a defense-in-depth philosophy: instead of relying only on embedding similarity, it layers clustering, centroid matching, refinement models, and data augmentation to reduce attack success rates with manageable computational impact. Future extensions may incorporate adaptive thresholding, online drift detection, and broader evaluations across diverse RAG workloads to further generalize the framework. Additionally, exploring adaptive attacks that specifically target cluster-centroid architectures, integrating anomaly detection mechanisms to identify suspicious query patterns, and investigating alternative similarity measures beyond cosine distance represent important directions for strengthening adversarial resilience in semantic caching systems.

Overall, these findings suggest that secure semantic caching requires richer semantic modeling than current embedding-based systems provide. By combining structural clustering, adversarial-aware refinement, and lightweight generative enrichment, SAFE-CACHE demonstrates a practical and effective approach for building resilient caching layers in modern RAG pipelines.

## Data Availability

The datasets generated and analyzed during this study are available from the corresponding author upon reasonable request.

## Material availability

The fine-tuned Gemma-3 1B model for intent-preserving paraphrase generation is publicly available at https://huggingface.co/mohanadffy/gemma3-intent-paraphraser-lora.
